# Supplementary material for: An Immobilized Form of a Blend of Essential Oils Improves the Density of Beneficial Bacteria, in Addition to Suppressing Pathogens in the Gut and Also Improves the Performance of Chicken Breeding
Source: Microorganisms. 2023 Jul 31;11(8):1960. doi: 10.3390/microorganisms11081960 (PMC10459846; doi:10.3390/microorganisms11081960)
Supplement: Supplementary file 1 [file microorganisms-11-01960-s001.zip › Fig. S1.pptx]

## Slide 1
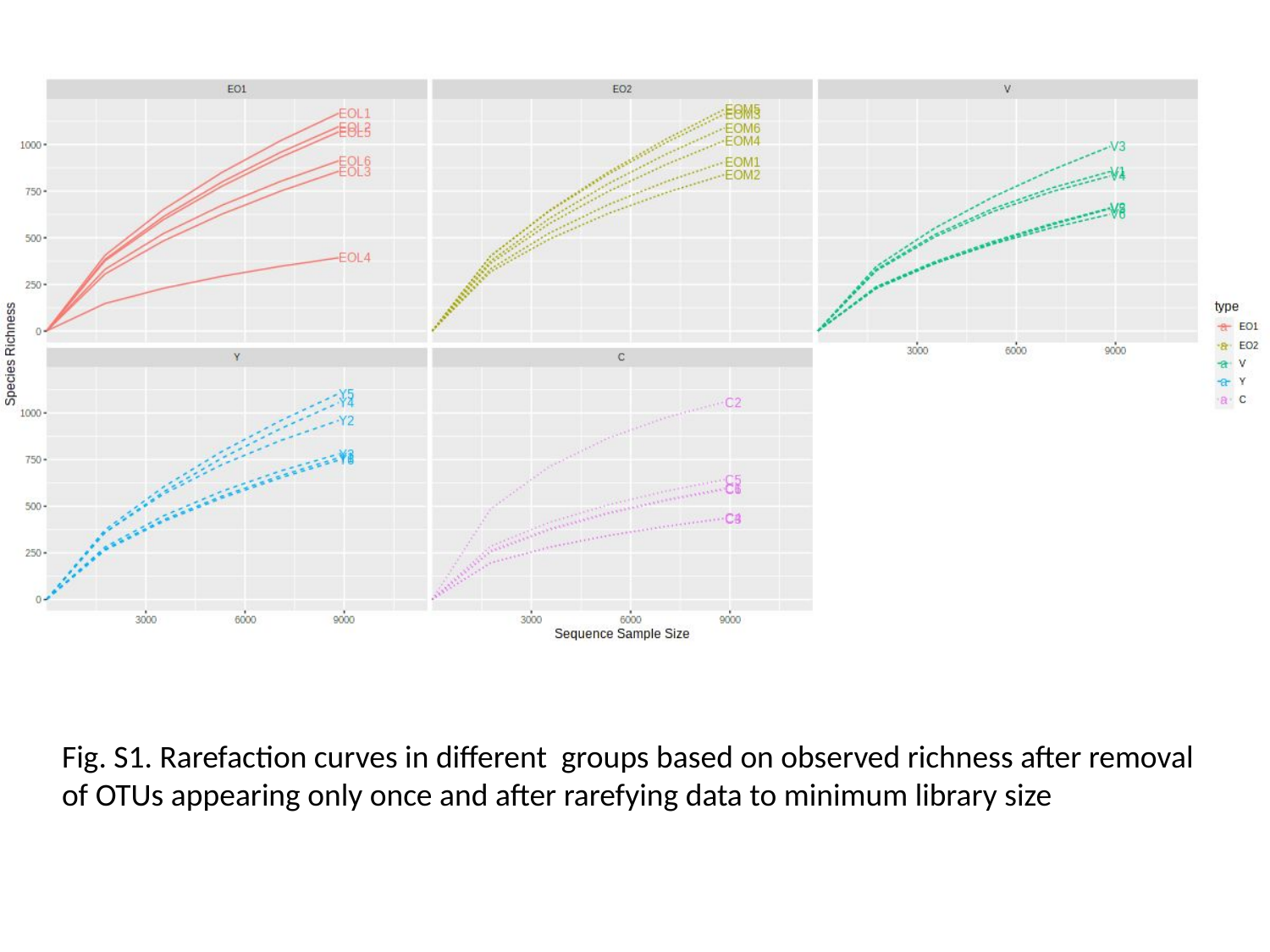

Fig. S1. Rarefaction curves in different groups based on observed richness after removal
of OTUs appearing only once and after rarefying data to minimum library size
