# Supplementary material for: An Immobilized Form of a Blend of Essential Oils Improves the Density of Beneficial Bacteria, in Addition to Suppressing Pathogens in the Gut and Also Improves the Performance of Chicken Breeding
Source: Microorganisms. 2023 Jul 31;11(8):1960. doi: 10.3390/microorganisms11081960 (PMC10459846; doi:10.3390/microorganisms11081960)
Supplement: Supplementary file 1 [file microorganisms-11-01960-s001.zip › Table S1.pdf]

Table S1. Ingredient and nutrient composition on basal Diet (as fed basis, %)

Ingredients Composition (g/kg)	Pre-Starter (d 1 - 14)	Starter (d 15 - 28)	Finisher (29 d onwards)
Maize	539.8	556.1	582.0
Soy bean meal	348.0	316.7	270.8
Meat and bone meal	39.7	39.7	39.7
Stone grit	24.5	10.6	10.7
Fat (Vegetable oil)	19.0	42.9	63.4
Dicalcium phosphate (DCP)	15.2	18.6	16.0
DL-methionine	3.05	2.65	2.67
L- Lysine HCl	1.35	0.750	1.12
L-Threonine	0.653	0.260	0.39
Common salt	4.0	4.1	4.0
Trace Mineral Mix [1]	1.225	2.0	2.5
AB2D3K [2]	0.204	0.360	0.375
B complex vitamin [3]	0.204	0.360	0.375
Choline chloride	1.02	2.0	2.5
Toxin binder (Bentonite)	1.02	2.0	2.5
Sodium bicarbonate	1.02	1.0	1.0
Nutrient Composition (%)			
Metabolizable energy (Kcal/kg) [4]	3000	3100	3250
Protein [5]	23.7	22.3	23.7
d Lysine[4]	1.28	1.15	1.06
d Methionine[4]	0.64	0.57	0.56
d TSAA[4]	0.95	0.87	0.83
d Threonine[4]	0.86	0.77	0.71
d Tryptophan[4]	0.27	0.25	0.23
Calcium[5]	0.96	0.87	0.88
Total Phosphorus[5]	0.75	0.70	0.68
Available Phosphorus[4]	0.48	0.44	0.44

1 composition (g/kg): manganese:110; zinc:100; copper:20; iron:110; selenium:1.5; iodine: 2.5; cobalt:1.0; chromium: 0.4;

2 composition (per g): retinolacetate: 82500IU; riboflavin: 50mg; cholecalciferol: 12000IU; menadione: 12000IU;

3 composition (mg/per g): thiamin:4; pyridoxin:8; cyanocobalamine:0.04; α-tocopherol:40; Calcium D Pantothenate:40; Niacin: 60; biotin:1.2;folic acid:4

4 Calculated

5 Analysed
