# Supplementary material for: An Immobilized Form of a Blend of Essential Oils Improves the Density of Beneficial Bacteria, in Addition to Suppressing Pathogens in the Gut and Also Improves the Performance of Chicken Breeding
Source: Microorganisms. 2023 Jul 31;11(8):1960. doi: 10.3390/microorganisms11081960 (PMC10459846; doi:10.3390/microorganisms11081960)
Supplement: Supplementary file 1 [file microorganisms-11-01960-s001.zip › Table S2.pdf]

Table S2. Effect of supplementing feed additives on apparent total tract digestibility (nutrient retention)

	**Total tract nutrient retention (g /kg)**		
	**DM**	**EE**	**CP**
V	78.09	91.8	88.2
C	76.09	91.6	88.8
Y	77.6	92.2	88.2
EO1	77.0	92.3	88.0
EO2	77.1	92.2	88.7
SEM	0.535	0.187	0.277
N	4	4	4
P value	0.154	0.089	0.254

C, control (basal diet); V; virginiamycin (40g/ton); Y, Yeast (S. cerevisiae, 25 billion CFU/g;500g/ton); EO1, Essential oil mix immobilized microspheres (200g/ton), EO2, Essential oil mix immobilized microspheres (400g/ton); P, probability; N, number of replicates; SEM, standard error mean
