# Supplementary material for: An Immobilized Form of a Blend of Essential Oils Improves the Density of Beneficial Bacteria, in Addition to Suppressing Pathogens in the Gut and Also Improves the Performance of Chicken Breeding
Source: Microorganisms. 2023 Jul 31;11(8):1960. doi: 10.3390/microorganisms11081960 (PMC10459846; doi:10.3390/microorganisms11081960)
Supplement: Supplementary file 1 [file microorganisms-11-01960-s001.zip › Table S3.pdf]

Table S3. Effect of additives on serum antioxidant enzymes, HI titre against Newcastle disease virus and cutaneous response to phytohaemaglutin P

Treatment	CMI Response(mm increase in skin thickness)	HI titer, log 2	LPX, (nmol MDA/mg)	GPX (units/ml)	GRX (units/ml)	SOD (units/ml)
V	0.415a	4.167	7.763	311.9b	1929	2.931
C	0.643a	2.667	8.042	327.1b	2011	3.410
Y	0.398a	3.667	8.752	281.1b	2012	4.141
EO1	1.018b	4.167	8.445	207.3a	2062	3.390
EO2	1.028b	4.333	8.103	210.5a	2011	4.601
SEM	0.055	0.212	0.149	8.96	26.76	0.220
P value	0.001	0.297	0.418	0.001	0.886	0.329

[abc] means having common superscript in a column do not vary significantly($P<0.005$) CMI cell mediated immunity; ND Newcastle disease; LPX Lipid Peroxidation; GPX Glutathione Peroxidase; GRX Glutathione Reductase; SOD Superoxide dismutase; C, control (basal diet); V; virginiamycin (40g/ton); Y, Yeast (S. cerevisiae, 25 billion CFU/g;500g/ton); EO1, Essential oil mix immobilized microspheres (200g/ton), EO2, Essential oil mix immobilized microspheres (400g/ton); P, probability; SEM, standard error mean
