# Supplementary material for: An Immobilized Form of a Blend of Essential Oils Improves the Density of Beneficial Bacteria, in Addition to Suppressing Pathogens in the Gut and Also Improves the Performance of Chicken Breeding
Source: Microorganisms. 2023 Jul 31;11(8):1960. doi: 10.3390/microorganisms11081960 (PMC10459846; doi:10.3390/microorganisms11081960)
Supplement: Supplementary file 1 [file microorganisms-11-01960-s001.zip › Table S4.pdf]

Table S4. Effects of treatments on abundance of *E.coli* and *Lactobacillus spp. in gut content*

**Group/parameter**	**E coli (log 10 values)**	**Lactobacillus (log 10 values)**	**Total bacteria(log10 values)**
V	2.78b	2.50c	9.22
C	2.48b	2.59c	9.49
Y	2.50b	2.65c	9.16
EO1	0.943a	0.906a	9.33
EO2	0.949a	1.62b	9.45
SEM	0.180	0.193	0.093
P Value	<0.0001	<0.0001	0.221
N	6	6	6

C, control (basal diet); V; virginiamycin (40g/ton); Y, Yeast (S. cerevisiae, 25 billion CFU/g;500g/ton); EO1, Essential oil mix immobilized microspheres (200g/ton), EO2, Essential oil mix immobilized microspheres (400g/ton); P, probability; SEM, standard error mean; means bearing common superscript does not differ significantly (P<0.05)
