# Supplementary material for: An Immobilized Form of a Blend of Essential Oils Improves the Density of Beneficial Bacteria, in Addition to Suppressing Pathogens in the Gut and Also Improves the Performance of Chicken Breeding
Source: Microorganisms. 2023 Jul 31;11(8):1960. doi: 10.3390/microorganisms11081960 (PMC10459846; doi:10.3390/microorganisms11081960)
Supplement: Supplementary file 1 [file microorganisms-11-01960-s001.zip › Table S5.pdf]

Table S5: Statistical analysis of alpha diversity measures

**Metrices**	**Mean values**					**Kruskal-Wallis test (P-value)**	**Dunn post hoc test –FDR adjusted P values**									
	**C**	**V**	**Y**	**EO1**	**EO2**		**C_V**	**C_Y**	**C_EO1**	**C_EO2**	**V_Y**	**V_EO1**	**V_EO2**	**Y_EO1**	**Y_EO2**	**EO1_EO2**
Richness																
Observed richness	625.8	769.5	901	916	1034	0.026	1.00	0.616	0.217	0.028	1.00	1.00	0.358	1.00	1.00	1.00
Chao1	936	1430	1809	1825	2126	0.007	1.00	0.059	0.059	0.012	1.00	1.00	0.663	1.00	1.00	1.00
ACE	1009	1618	2055	2102	2469	0.003	1.00	0.043	0.048	0.004	1.00	1.00	0.304	1.00	1.00	1.00
Diversity																
Simpson	0.896	0.888	0.930	0.876	0.963	0.139										
Shannon	3.98	3.81	4.12	4.01	4.63	0.113										
Fisher	159	204	253	264	306	0.026	1.00	0.616	0.217	0.028	1.00	1.00	0.358	1.00	1.00	1.00

C, control (basal diet); V; virginiamycin (40g/ton); Y, Yeast (S. cerevisiae, 25 billion CFU/g;500g/ton); EO1, Essential oil mix immobilized microspheres (200g/ton), EO2, Essential oil mix immobilized microspheres (400g/ton)
