# Supplementary material for: An Immobilized Form of a Blend of Essential Oils Improves the Density of Beneficial Bacteria, in Addition to Suppressing Pathogens in the Gut and Also Improves the Performance of Chicken Breeding
Source: Microorganisms. 2023 Jul 31;11(8):1960. doi: 10.3390/microorganisms11081960 (PMC10459846; doi:10.3390/microorganisms11081960)
Supplement: Supplementary file 1 [file microorganisms-11-01960-s001.zip › Table S6.pdf]

Table S6: Statistical analysis of beta diversity measures for comparing microbial community composition among different groups (PERMANOVA and PERMDISP results along with PCoA/NMDS parameters).

					PCoA		NMDS	
**Taxonomic level**	**Diversity metric**	**PERMANOVA**			**% variation explained at PC1**	**% variation explained at PC2**	**Stress**	**PERMDISP-P-value**
		**Pseudo-F**	**R^2**	**P-value**				
OTU	Bray-Curtis	3.44	0.355	<0.001	24.4	19.9	0.1048	0.519
	Jaccard	2.45	0.282	<0.001	16.7	15.1	0.1048	0.492
	Jensen-Shannon	5.61	0.473	<0.001	36.1	26.1	0.1013	0.580
Phylum	Bray-Curtis	2.85	0.313	0.009	55.1	42.2	0.1165	0.877
	Jaccard	2.89	0.316	0.006	50.0	39.0	0.1165	0.618
	Jensen-Shannon	5.28	0.458	<0.001	78.9	41.1	0.0450	0.218
